# The Effect of Place Attachment on Overseas Students’ Tourism Ambassador Behavior: A Mediation Role of Life Satisfactionrdrd

**DOI:** 10.3389/fpsyg.2021.766997

**Published:** 2021-12-13

**Authors:** Xin Wang, Ivan Ka Wai Lai, Xinyu Liu

**Affiliations:** Faculty of International Tourism and Management, City University of Macau, Macao, Macao SAR, China

**Keywords:** place attachment, life satisfaction, human-place emotion connection, ambassador behavior intention, word of mouth, overseas students

## Abstract

Life satisfaction is a research hotspot in positive psychology in recent years. This study uses overseas students as subjects and attempts to examine the effect of place attachment and student life satisfaction on Mainland Chinese students’ word-of-mouth (WOM) recommendations and their Ambassador Behavioral (AB) intention. A survey was systematically conducted in six institutions in Macao. The results of 312 valid data indicate that place dependence has a positive influence on place identity; place identity and place dependence have a positive influence on student life satisfaction; student life satisfaction mediates the influence of the two dimensions of place attachment on WOM and AB intention. Recommendations are provided to improve overseas students’ life satisfaction in the study places. It helps to improve their sense of ownership and actively participate in the construction of the study places.

## Introduction

With the development of the economy and the influence of globalization, population mobility increases, and the relationship between people and places becomes a prominent problem. Place attachment is one of the most important theories to explain the relationship between human and place (Lewicka, 2011). It originated from psychological research. With the deepening of research, place attachment theory has gradually become a research hot spot in human geography, environmental psychology, and sociology. Previous studies in tourism have used place attachment theory to understand the relationship between visitors or residents and destinations. Place attachment is a connection formed by the interaction between people and places. Its essence is a theoretical framework with three dimensions: people, psychological process and place (Jorgensen and Stedman, 2006). Sojourners have a long-term destination experience and prolonged social contact with the host region (Brown, 2009). Overseas students are the most representative group of sojourners. They travel to other places to study and stay there a long time. They have a full understanding and special feelings about the place of study and become an important force to support the development of study place, so they may take a city tourism ambassador role (Chen et al., 2015). However, it remains unclear what is the underlying mechanism behind the formation of such positive behavioral intention. Since people’s emotional attachment to a place is closely related to a sense of satisfaction that the place brings to people (Shumaker and Taylor, 1983), it is essential to explore the emotional connection of overseas students to their study places to enhance their life satisfaction and urban development.

A positive correlation between satisfaction and behavioral intention has been demonstrated in many previous studies (Kotler et al., 1993) that includes the tendency to recommend a destination to other people (Yoon and Uysal, 2005). However, overseas students are different from general tourists because they stay in one destination for a certain period. Therefore, their satisfaction should not be seen as a one-time transactional travel experience but rather as satisfaction with student life in the city where they study and live. Cognitive congruence theory suggests that attitude is an important driver of behavior, and many previous studies have shown that individuals are more likely to behave responsibly toward an object if they have a positive attitude toward it (Zhang and Xu, 2019). Therefore, in the context of this study, it is reasonable to predict that students’ life satisfaction with a destination of study, as a significant attitudinal factor, will impose a positive effect on their destination brand-building behavior.

This study aims to examine the inter-relationship between place attachment, student life satisfaction, and destination brand-building behaviors of overseas students. The literature on tourism reveals that the two major components of place attachment are “place identity” and “place dependence” (Yuksel et al., 2010; Lee et al., 2012). From another perspective, destination brand-building behavior is mainly composed of “word of mouth” (Chen et al., 2018) and “ambassador behavior” (Wassler et al., 2021). Therefore, the conceptual model of this study includes these four elements. This study makes considerable contributions to the literature in two ways. First, the theory of place attachment is used to explain the human-place bonding (Ramkissoon and Mavondo, 2015), and the need satisfaction theory is used to explain the cognitive psychological factors caused by people’s deep behaviors (Pagán, 2015). The two concepts have rarely been empirically linked as potential factors to explain resident and tourist behavior. This study combines place attachment theory and life satisfaction theory to explain the formation of overseas students’ ambassadorial behavior. Second, the majority of previous studies on word-of-mouth (WOM) and ambassadorial behavior in destination branding have focussed on examining local residents and tourists (Litvin et al., 2008; Chen and Šegota, 2015). Sojourners have a long-term destination experience and prolonged social contact with the host region (Brown, 2009). However, there is little research on the potential of their direct involvement in destination marketing and the psychological factors that influence their destination building behavior. Therefore, this study contributes to destination brand research by adding findings on overseas student behaviors in tourism marketing. The study has implications for the destinations’ governments with reference to understanding how to involve major groups of sojourners as overseas students in the promotion of tourist destinations where they studied and lived.

## Literature Review

### Overseas Students in Tourism

At present, most studies on overseas students focus on education and culture, mainly discussing their life experiences and cross-cultural adaptation (Sung, 2020). In the tourism field research, overseas students are generally considered to be an important tourism market, and their tourist behavior and reception of friends and relatives during the study period as well as their economic contribution to the places of study has been confirmed (Gardiner et al., 2013). Previous studies have determined tourist and resident’s WOM recommendations and ambassador behavioral intentions (Lai et al., 2018; Wassler et al., 2021). However, the studies on the destination brand-building behavior of sojourners are rare. Overseas students are a typical example of sojourners. From the perspective of tourism destination marketing, it is essential to examine Mainland Chinese students’ intention to participate in the branding of the destination. Xu and Huang (2018) mentioned that the life experience of students in mainland China was an important motivation for them to revisit, but they did not have an in-depth discussion on students’ life characteristics and life satisfaction from the perspective of a human–place relationship. Therefore, it makes sense to explore their current sense of place and study how life affects their willingness to act as a “middleman” and “facilitator,” and as those who recommend cities as future tourist destinations.

### Student Life Satisfaction

Life satisfaction is a long-term cognitive perception (Bartram, 2015), it refers to the overall quality of an individual’s life (Pagán, 2015). With the development of social cognition and cognitive psychology, affective factors, such as those influencing satisfaction evaluation, have become increasingly important in satisfaction research (Bradshaw et al., 2011; Gavín-Chocano et al., 2020). To a certain extent, these affective factors can be explained or predicted to a considerable extent; these include use behavior, complaining behavior, word of mouth and repurchase plan, and so forth (Westbrook, 1987). Oliver (1993) explained the determinants and formation of emotion, in terms of satisfaction evaluation, and positive emotions like being interested and pleased, affect the formation of satisfaction. Consequently, a satisfied person tends to recommend a destination to other people. Unanue et al. (2017) observed that satisfaction in one domain positively influences other life domains, as well as overall life satisfaction. Ramkissoon et al. (2018) also supported that place satisfaction influences the quality of life of repeat visitors to a national park.

In tourism research, a tourist’s overall satisfaction refers to a subjective evaluation of all preceding travel experiences to a destination. Unanimously, the overall satisfaction of tourists positively affects their tendency to recommend a destination to other people (Yoon and Uysal, 2005), which may mean that satisfied visitors hold positive attitudes toward the destination. Therefore, while the studies focussed on the “affective,” researchers used overall satisfaction based on all past visits to a destination to evaluate the past travel experience (Al Azmeh, 2019).

Based on the literature research, the studies of life satisfaction are focused on adults (Chen et al., 2014), while in the context of higher education, students are treated somewhat differently. Specifically, student life includes studying and local living activities. For example, they would visit local enterprises and become local volunteers. They would go out for dinners with friends every week and join local festivals with the local residents together during the holidays. In general, active and in-depth social participation can enhance individual and collective control, self-efficacy, and clearer life goals, thereby affecting citizens’ life satisfaction (Budge et al., 2019). For residents, their quality of life can be measured by their desirable level and enjoyable level of the place to live (Aleshinloye et al., 2021). For college students, the quality of life and satisfaction can be measured by eight aspects: emotional, physical, material, personal developmental, self-determination, interpersonal quality of relationships, social tolerance, and rights (Schalock et al., 2008). In addition, Benjamin (1994) also pointed out that some unique life areas, including “academic,” “university service,” and “university management,” including on-campus and off-campus life experiences, can also affect students’ life satisfaction (de Róiste et al., 2012). Furthermore, research also shows that participation in school activities has a greater impact on students’ life satisfaction (Gempp and González-Carrasco, 2021). Students who actively participate and identify with the school at a higher level are more capable of establishing relationships with others, as well as supporting and cooperating with them (Chen et al., 2015). They can gain better self-esteem and greater autonomy and self-control through the process of participation. These experiences and feelings thereby increase life satisfaction and lower the possibility of causing anti-social or dangerous behaviors (Finn and Rock, 1997).

In recent years, the life satisfaction of overseas student groups has attracted much attention. The study of Xu et al. (2020) has explored the relationship between Mainland Chinese student life satisfaction and the motivation of destination marketing. In the study by Xu and Huang (2018), on length of stay, students may be classified as temporary residents (Glover, 2011) or Mainland visitors (Xu and Huang, 2018). Simpson et al. (2016) found migrant tourists were more satisfied with the destination as the length of residence time increases, and this affected the likelihood of recommending the destination to someone else. Therefore, for Mainland Chinese students, their life satisfaction with the study place may influence their ambassador behavior. However, other than the length of residence time, researchers have not identified the factors related to student life satisfaction with the destination. Given that there are a large number of Mainland Chinese students studying abroad, it is important to explore the opportunities these students have to make a contribution to the destination branding process. It is what makes them feel satisfied with their studies and lives in the place of study.

### Place Attachment

In psychology, attachment is regarded as an adaptive emotional response and the state of a person in a specific social relationship (Huang and Zheng, 1991), initially considered to be the emotional connection between the baby and the caregiver (Bowlby, 1982). This was later referred to as a unique compound of emotions combined with positive emotions such as pleasure, happiness, and so forth (Huang and Zheng, 1991). With an expansion in the field of applied psychology, the concept of “place” received attention from environmental psychology, as the object of attachment (Hidalgo and Hernandez, 2001). It belongs to a kind of human-space relationship (Ramkissoon and Mavondo, 2015), which explored “Topophilia” and “sense of place.” Therefore, place attachment can be defined as a positive emotional bond between the individual and the place (Yuksel et al., 2010).

In the early literature, most attention was focused on the distinction between a functional bond and an emotional bond (Lin and Lockwood, 2014). “Place dependence” refers to a function that is satisfied in order to support the desired goal, which is the functional bond dimension (Anton and Lawrence, 2014). “Place identity” is a symbolic feeling that combines the image of a place with the distinction of self, and this is the emotional bond dimension (Ujang and Zakariya, 2018). This two-dimensional structure has become a matter of consensus in the academic world. Although some scholars have extended the dimensionality of place attachment (Ramkissoon et al., 2013, 2018; Isa et al., 2020), the validity and universality of these two dimensions have been verified in different situations (Brown et al., 2015). Therefore, this study also adopts these two dimensions to measure the place attachment of Mainland Chinese students.

In the place attachment research, some researchers identified the antecedents of place attachment. For example, Aleshinloye et al. (2021) indicated that resident empowerment and quality of life positively impact the place attachment of residents. Van Riper et al. (2019) argued the motivations (achievement, similar people, learning, enjoying nature, and escape) are antecedents of visitors’ place attachment in Australian national park. However, more research tended to study the influence of place attachment on the attitudes and behaviors of residents and tourists. For example, Ramkissoon (2020a,2021) proposed place attachment is a way to support residents’ wellbeing in the crisis of COVID-19. For tourists, place attachment also influences tourists’ revisitation (Isa et al., 2020; Majeed and Ramkissoon, 2020).

In the literature on brand citizenship behaviors, it is helpful to understand the potential causal relationships between human-place relationships and behavioral intentions (Chen et al., 2018). In this sense, place attachment—specifically, its dimension of place identity (reflecting one’s identity and perceived citizenship)—may have an impact on place-related behaviors such as WOM and goodwill ambassador behaviors. However, how to strengthen the influence of place attachment on place related behaviors is not well studied, so this study attempts to fill the research gap and explore the role of student life satisfaction for Mainland Chinese students in building the destination brand regarding the place attachment theory.

### Destination Brand-Building Behaviors

Destination branding is a series of efforts made by a country, industry organization, or responsible government agency to promote the place represented (Morhart et al., 2009). Destination branding literature first surfaced in 1996; an academic postulated that destinations are the biggest brand in the travel industry and that in the future marketing would be a brand battle (Chen and Šegota, 2015). In the context of tourism, the name of the place is not enough to build its brand. For the majority of destinations, slogans are a necessary form of public expression of a destination’s brand positioning strategy, but they fail to achieve anything other than an ephemeral indifference. Residents can be considered as employees who advocate the destinations to their friends and families (King et al., 2015). This behavior can be regarded as a destination’s brand-building. In general, brand-building behaviors can be divided into two forms, “in-role” and “extra-role,” according to the degree of contribution required by the actors.

In-role brand-building behavior refers to the fact that residents can serve as goodwill ambassadors, who perceive the destination brand consistently with how the destination conveys itself through public messages and marketing communications; such as residents’ support for their participation and involvement, as well as tourism planning and development (Morhart et al., 2009). The behaviors of these residents constitute ambassador behavior which is a development-related behavior aimed to enhance the equity of a destination brand (Hung et al., 2017). Extra-role brand-building behavior, in the context of tourism destination, refers to word-of-mouth (WOM), and discretionary and individually generated actions. Moreover, WOM is becoming the most powerful form of marketing in the new information age (Simpson and Siguaw, 2008).

Overseas students including the Mainland Chinese students coming to a tourist destination, who have a long-term destination experience, for a period of at least 6 months, can be defined as sojourners, and these are similar to local residents (Liu and Ryan, 2011). Local residents’ hosting experience and attitude to the destination can be accumulated in the leisure space, which will influence the visitors’ experiences. With the experience of cultural adjustment and the increase of residence time, the Mainland Chinese students may also exhibit destination brand-building behaviors to guide their friends and family thus influencing them to visit the study place.

## Methodology

### Research Hypotheses

Moore and Graefe (1994) pointed out that people need more time to develop a personal sense of identification with a place than to have a personal sense of dependence on a place. Therefore, a cause-and-effect relationship exists between place dependence and place identity (Vaske and Kobrin, 2001). Researchers have verified this relationship that place identity is an intermediary in the influence of place dependence on behavioral intention (Su and Hsu, 2019).

Wang and Davidson (2008) claimed that there is a semi-stable human-place relationship between Mainland Chinese students and the study city. Thus, the initial formation of Mainland Chinese students’ attachment to the study city is mainly due to its specific physical and social environment and continuous participation in social activities (Chen et al., 2021). This is physical dependence on the place. Subsequently, the Mainland Chinese students perceive emotional identification with the study city. Accordingly, it can be assumed that for sojourners, place dependence is an antecedent of place identity. Thus, this study proposes the following hypothesis.

**Hypothesis 1:** Mainland Chinese students’ place dependence has a positive impact on their place identity.

From the perspective of the human-place bond, people attached to their living place is bound to exert a certain significant influence on their life satisfaction. Fleury-Bahi et al. (2008) proposed that intimate human-place dependence, in short, place attachment, offers a unique perspective of people’s own perceptions, including their psychological perceptions. For tourists, in Hwang et al. (2005) study of Taiwanese national parks, it was confirmed that the existence of place attachment could influence tourists’ satisfaction. Ramkissoon et al. (2018) in their study of visitors at the Dandenong Ranges National Park, in Australia, also supported the existence of place attachment on quality of life. On the other hand, Ramkissoon (2020a,2021) recently argued that place attachment influences residents’ wellbeing in the crisis of COVID-19. Since place attachment has an intensified connection to satisfaction (Qiu et al., 2020), so it is possible that a sense of attachment and belongingness of Mainland Chinese students may influence their life satisfaction. Thus, this study proposes the following hypotheses.

**Hypothesis 2a**: Mainland Chinese students’ place identity positively affects their life satisfaction.

**Hypothesis 2b**: Mainland Chinese students’ place dependence has a positive impact on their life satisfaction.

The self-determination theory (SDT) argues that employees are likely to internalize the brand-based role identity when they experienced the satisfaction of their needs (Hancox et al., 2015). The more satisfied consumers are more likely to exhibit positive brand-building behaviors (Yoon and Uysal, 2005). In tourism research, Khuong and Van Nga (2018) argued that tourists’ satisfaction with the destination would influence their intention to perform positive brand-building behaviors. The Mainland Chinese students act as internal employees of the company: once they are satisfied with the destination they may exhibit destination brand-building behaviors. Because destination brand-building behaviors include WOM and ambassador behavior, the study proposes the following hypotheses regarding the brand-building behaviors:

**Hypothesis 3a**: Mainland Chinese students’ life satisfaction has a positive impact on their word-of-mouth intention.

**Hypothesis 3b**: Mainland Chinese students’ life satisfaction has a positive impact on their ambassador behavior.

A high level of attachment to a place can result in positive residents’ behaviors, such as protecting the environment (Ramkissoon, 2020b) and spreading a positive destination brand (Ahearne et al., 2005), because place attachment can motivate individuals to protect and improve it. As a resident’s attachment increases, he or she is more likely to build the destination’s brand (Chen et al., 2018). Hung et al. (2017) found that the highly attached residents would engage in brand-building activities. Thus, this study proposes the following research hypotheses:

**Hypothesis 4a**: Mainland Chinese students’ place identity positively affects their word-of-mouth intention.

**Hypothesis 4b**: Mainland Chinese students’ place identity positively affects their ambassador behavior.

**Hypothesis 5a**: Mainland Chinese students’ place dependence positively affects their word-of-mouth intent ion.

**Hypothesis 5b**: Mainland Chinese students’ place dependence positively affects their ambassador behavior.

**FIGURE 1:**
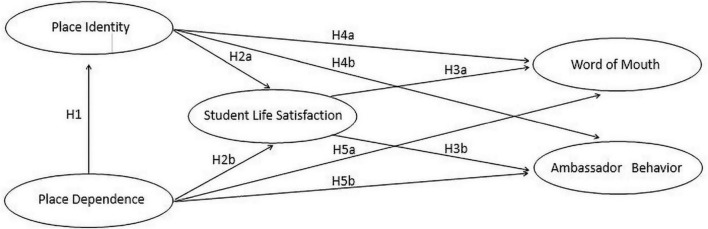
Research models.

The research model is shown in Figure 1.

### Research Setting

Overseas students are an important group of sojourners. They live temporarily in other places due to their established learning goals. Overseas students usually transfer the academic knowledge and life experience acquired in their sojourn places to their host countries to promote the flow of international knowledge and information. The number of Mainland Chinese students is the largest among overseas students. Macao is the tenth most popular study destination for Mainland Chinese students and received around 200,000 students between 1998 and 2018 (UNESCO, 2019). According to the latest statistics, there were 18,904 Mainland Chinese students in Macao. This is a ratio of 1:37 in relation to Macao’s total population (Macao Higher Education Bureau, 2020). Therefore, choosing Macao as a research site can effectively demonstrate the influence of Mainland Chinese students in destination brand-building behaviors.

Macao is located in the Pearl River Delta on the South-Eastern coast of China, about 60 km east-northeast of Hong Kong. Macao has a total area of 32.9 square kilometers and a population of about 682,800 people. The majority of Macao’s residents are Chinese, accounting for over 90% of the total population, while the rest are Portuguese, Filipino, and people of other nationalities. Macao is an international free port, a world center for tourism and leisure, and one of the world’s four major gambling cities. Although Macao is small in terms of area, it has many world heritage sites such as A-Ma Temple, Yapo Well, and the Ruins of the Cathedral of Saint Paul, as well as several scenic spots such as museums, churches and temples. The historic district of Macao was included in the World Cultural Heritage List in 2005. At the same time, Macao has also been named by UNESCO as the “City of Creative Cuisine.” Macanese dishes are one of Macao’s unique specialities, and the culinary skills of Portuguese natives were added to the list of Macao’s Intangible Cultural Heritage assets in 2012.

### Measurement Scales and Questionnaire Design

The measurable items for place dependence, place identity, and student life satisfaction are derived from Huang and Hsu (2009), Chen and Dwyer (2018), and Aleshinloye et al. (2020), respectively. For measuring destination brand-building behaviors, three items for WOM and three items for ambassador behavior are derived from Chen and Dwyer (2018). For measurement, participants responded using a 7-point Likert scale ranging from 1 (strongly disagree) to 7 (strongly agree). Table 1 lists the 19 items of the research model. An English questionnaire was translated into Chinese and back translated into English to eliminate the translation bias. Two professors in the field of tourism helped to validate the content of the Chinese and the English versions. Since the target samples in this study are Mainland Chinese students, a pilot test with 50 of them was conducted in Macao at the end of October 2020 to further confirm the content. This process allowed for the wording of some of the items of the questionnaire to be improved. One measurement item—“Macao means a lot to me”—was deleted due to its lower reliability.

**TABLE 1:** Measurement scales.

	Measured item
	Place dependence (PD)
PD1	For my learning, the resources and facilities provided by Macao are the best.
PD2	For my learning and life, I could not imagine anything better than the resources and facilities provided by Macao.
PD3	I enjoy living in Macao and its social environment more than any other cities.
PD4	I really miss Macao when I am away from it for too long.
	Place identity (PI)
PI1	I feel the Macao is a part of me
PI2	I identify strongly with Macao
PI3	Living in Macao says a lot about who I am
PI4	The Macao is very special to me
PI5	I am attached to the Macao
	Word of mouth (WOM)
WOM1	When “talk up” Macao, I often speak favorably about Macao to people I know.
WOM2	I bring up Macao in a positive way in conversations I have with friends and acquaintances.
WOM3	On social media, I often post positive information about Macao.
	Ambassador behavior (AB)
AB1	In tourist contact situations, I ensure that my personal appearance is in line with Macao sider’s appearance in my mind.
AB2	I adhere to my standards for Macao sider’s behavior.
AB3	I see that my actions in tourist contact are not at odds with a Macao sider’s behavior.
	Student life satisfaction (SLS)
SLS1	My overall evaluation of the experience of studying and living in Macao is positive.
SLS2	My overall evaluation of the experience of studying and living in Macao is favorable.
SLS3	I am satisfied with my experience of studying and living in Macao.
SLS4	I am pleased with my experience of studying and living in Macao.

The questionnaire has three sections. The first question is a filter question: “Are you a student of a Macao higher education institution from Mainland China?” This was asked to ascertain whether or not the respondent qualified for the study. Only those responding “yes” to the question were invited to complete the questionnaire. The second section questions were designed to measure the five constructs. The third section is the background information of respondents.

### Data Collection

According to 2019 statistics from the Macao Higher Education Bureau, there are 10 institutions of higher learning in Macao, six of which have approved the admission of mainland students. The proportion of mainland students in the six schools is as follows: Macao University of Science and Technology: 56.5%, University of Macao: 19.3%, City University of Macao: 19.0%, Macao Polytechnic Institute: 3.0%, Macao Institute of Tourism: 2.0%, and Kiang Wu Nursing College of Macao: 0.2%. The questionnaires were distributed according to the above proportion.

From 23rd to 27th November 2020, six research assistants administered the survey at five locations (the school gate, the library, the dining room, the student dormitory building, and the teaching building) one by one in six institutions from 11:00 to 19:00 every day. Data were systematically collected. The research assistants chose one in 10 people who passed by to fill in the questionnaire. If the respondent was not a Mainland Chinese student or refused to answer the questionnaire because of other reasons, the research assistants would count another 10 people. Four hundred samples were collected. Since some students gave similar rates for most items, 88 questionnaires were removed. Table 2 reports the respondents’ profiles. There were 114 males (36.5%) and 198 females (63.5%). The characteristics of the samples matched the population of Mainland Chinese students in Macao. One-third of them stayed less than 1 year, 1–2 years, and over 2 years.

**TABLE 2:** Sample profile (*n* = 312).

		Frequency	Percent
Gender	Males	114	36.5%
	Females	198	63.5%
Age	18–22	241	77.3%
	23–27	64	20.5%
	28–32	5	1.6%
	Over 32	2	0.6%
Education (Current state)	Advanced placement	3	1.0%
	Bachelor	225	72.1%
	Master	71	22.8%
	Doctor	13	4.2%
Length of stay (Years of studying and living in Macao)	1 Year or less	98	31.4%
	2 Years	98	31.4%
	3 Years	64	20.5%
	4 Years or Over	52	16.7%
College and University	Macao University of Science and Technology	164	52.6%
	University of Macao	62	19.9%
	City University of Macao	63	20.2%
	Macao Polytechnic Institute	11	3.5%
	Macao Institute of Tourism	9	2.9%
	Kiang Wu Nursing College of Macao	1	0.3%
	Others	2	0.6%
Mainland Region of China	South China	85	27.2%
	East China	92	29.5%
	Central China	33	10.6%
	North China	23	7.4%
	Northwest China	14	4.5%
	Southwest China	42	13.5%
	Northeast China	23	7.4%

## Results

### Outer Model Analysis

Table 3 reports the descriptive statistics of 19 items. The minimum value of the PLS factor loading is 0.735 (>0.700). All Cronbach’s α values are higher than the threshold 0.7 and all composite reliability (CR) values are higher than the threshold 0.7 too (as shown in Table 4; Henseler et al., 2015). According to Hair et al. (2010), the values of the average variance extracted (AVE) for each construct are above the recommended 0.5. It shows good reliability and convergent validity. Discriminant validity is examined by comparing the square root of AVE for each construct with the correlations between pairs of latent variables (Fornell and Larcker, 1981). Furthermore, all Heterotrait–Monotrait (HTMT) correlations are less than 0.90 (Henseler et al., 2015), achieving a satisfactory result.

**TABLE 3:** Descriptive statistics and factor loadings.

	Mean	*SD*	Kurtosis	Skewness	Loadings
PD1	5.276	1.029	0.411	–0.413	0.735
PD2	4.285	1.450	–0.260	–0.221	0.780
PD3	4.968	1.384	–0.492	–0.380	0.861
PD4	4.801	1.318	–0.285	–0.229	0.817
PI1	4.676	1.439	–0.296	–0.334	0.860
PI2	4.728	1.377	–0.044	–0.425	0.904
PI3	4.042	1.355	–0.051	–0.208	0.838
PI4	5.138	1.252	0.468	–0.617	0.845
PI5	4.984	1.223	0.244	–0.434	0.870
WOM1	5.147	1.140	0.129	–0.370	0.927
WOM2	5.253	1.125	0.492	–0.525	0.929
WOM3	5.237	1.098	0.128	–0.321	0.876
AB1	4.753	1.251	0.016	–0.374	0.860
AB2	4.279	1.353	0.165	–0.494	0.861
AB3	4.904	1.175	0.164	–0.383	0.834
SLS1	5.240	1.102	1.485	–0.762	0.900
SLS2	5.163	1.087	0.578	–0.570	0.936
SLS3	5.221	1.083	1.228	–0.617	0.926
SLS4	5.343	1.095	0.980	–0.595	0.920

**TABLE 4:** Reliability, construct validity, and correlation.

	Cronbach’s alpha	CR	AVE	Fornell-larcker criterion					Heterotrait-monotrait ratio				
				AB	SLS	PD	PI	WOM	AB	SLS	PD	PI	WOM
Ambassador behavior (AB)	0.811	0.888	0.725	0.851									
Student life satisfaction SLS)	0.940	0.957	0.847	0.645	0.920				0.732				
Place dependence (PD)	0.812	0.876	0.639	0.618	0.649	0.799			0.760	0.735			
Place identity (PI)	0.915	0.936	0.746	0.639	0.680	0.782	0.864		0.739	0.733	0.890		
Word-of-mouth (WOM)	0.897	0.936	0.830	0.638	0.686	0.651	0.675	0.911	0.744	0.748	0.752	0.744	

*AVE, average variance extracted; CR, construct reliability, Italic front-square-root of AVE.*

### Inner Model Analysis

**FIGURE 2:**
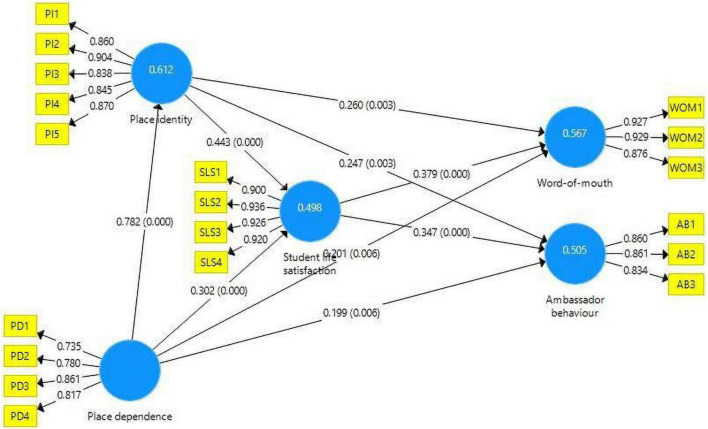
Results of PLS-SEM analysis.

Figure 2 shows the results of structural equation modeling (SEM) analysis. The analytical results indicate that student life satisfaction (SLS) has significant influences on Ambassador Behavior (AB) (β = 0.347, *p*-value < 0.001); SLS has significant influences on Word of Mouth (WOM) (β = 0.379, *p*-value < 0.001); Place Dependence (PD) has influences on AB (β = 0.199, *p*-value < 0.01); PD has significant influences on SLS (β = 0.302, *p*-value < 0.001); PD has significant influences on Place Identity (PI) (β = 0.782, *p*-value < 0.001); PD has influences on WOM (β = 0.201, *p*-value < 0.01); PI has influences on AB (β = 0.247, *p*-value < 0.01); PI has significant influences on SLS (β = 0.443, *p-*value < 0.001); PI has influences on WOM (β = 0.260, *p*-value < 0.01) The R^2^ of SLS, PI, WOM, and AB are higher than 0.25. Results indicate that all the nine hypotheses proposed were supported. Findings of the path relationships are displayed in Table 5. The multicollinearity was tested by variance inflation factor (VIF), all VIF values did not exceed 5, indicating an absence of multicollinearity issue (Hair et al., 2011).

**TABLE 5:** Results of hypotheses testing.

	Coefficient	T-statistics	f-Square	VIF	Test results
H1 PD→PI	0.782	34.941	1.577	1.000	Supported
H2a PI→SLS	0.443	5.889	0.152	2.577	Supported
H2b PD→SLS	0.302	4.069	0.071	2.577	Supported
H3a SLS→WOM	0.379	4.847	0.166	1.992	Supported
H3b SLS→AB	0.347	5.096	0.122	1.992	Supported
H4a PI→WOM	0.260	2.955	0.053	2.969	Supported
H4b PI→AB	0.247	2.965	0.041	2.969	Supported
H5a PD→WOM	0.201	2.722	0.034	2.760	Supported
H5b PD→AB	0.199	2.752	0.029	2.760	Supported

### Mediating Effect on Place Identity and Student Life Satisfaction

The results summarized in Table 6 show that both PI and SLS have significant mediating effects. Firstly, SLS mediates the positive impact of PD on AB (indirect effect = 0.105, *p*-value < 0.001) and WOM (indirect effect = 0.115, *p*-value < 0.01); SLS mediates the positive impact of PI on AB (indirect effect = 0.154, *p*-value < 0.001) and WOM (indirect effect = 0.168, *p*-value < 0.001). Continuing, PI mediates the positive impact of PD on AB (indirect effect = 0.193, *p*-value < 0.01) and WOM (indirect effect = 0.203, *p*-value < 0.01); PI mediates the positive impact of PD on SLS (indirect effect = 0.347, *p*-value < 0.001). Lastly, the mediating effect of both PI and SLS on the positive impact of PD on AB (indirect effect = 0.120, *p* < 0.001) and WOM (indirect effect = 0.131, *p* < 0.001) was found to be significant.

**TABLE 6:** The mediation effect of place identity and life satisfaction.

	Effect	*T-*value	2.5%	97.5%	Mediation
PD→SLS→AB	0.105	3.178	0.047	0.176	Partial mediation
PI→SLS→AB	0.154	3.653	0.080	0.245	Partial mediation
PD→PI→SLS→AB	0.120	3.580	0.062	0.195	Partial mediation
PD→PI→AB	0.193	2.889	0.065	0.330	Partial mediation
PD→PI→SLS	0.347	5.573	0.229	0.471	Partial mediation
PD→SLS→WOM	0.115	2.791	0.043	0.204	Partial mediation
PI→SLS→WOM	0.168	4.003	0.091	0.255	Partial mediation
PD→PI→SLS→WOM	0.131	3.922	0.070	0.202	Partial mediation
PD→PI→WOM	0.203	2.902	0.066	0.343	Partial mediation

## Discussion and Conclusion

### Conclusion

The results show that place dependence has a positive influence on place identity, and both place identity and place dependence have a positive effect on student life satisfaction. First, the results are consistent with previous studies (Moore and Graefe, 1994; Su and Hsu, 2019) where place dependence influences place identity, which implies that Mainland Chinese students have a personal sense of dependence and developed a connection with Macao after a period of studying and living there. Second, the results are also consistent with the results of Qiu et al.’s (2020) study in which Mainland Chinese students’ satisfaction with life in Macao is affected through the use of resources, facilities, and contact with the social environment during their study and life in Macao. The results of this study also find that two dimensions of place attachment and student life satisfaction influence Mainland Chinese students’ WOM and ambassador behavior. These findings are consistent with a previous study (Chen and Dwyer, 2018) in which place attachment is found to influence destination brand-building behavior, but their target was residents. And it implies that satisfied mainland Chinese students would construct positive brand-building behavior.

### Contributions

First, this study uses the theory of place attachment to explore the inter-relationship between place dependence, place identity, and overseas students’ life satisfaction. Although previous studies have confirmed the effect of place attachment on residents’ quality of life (Ramkissoon et al., 2018), the effect of place attachment on sojourners is still under research. Place attachment is an attitude toward the place, and attitude theory holds that attitude is composed of cognition, emotion and intentionality (behavior). The cognitive component of place attachment is the attitude, judgment, belief, value, and symbolic meaning of place based on the cognitive process of place perception and memory. Through memory, people can generate local meaning and associate it with themselves, forming local identity. As life satisfaction is positive psychology, this local meaning improves one’s wellbeing and overall life satisfaction (Ramkissoon, 2020a). For mainland Chinese students, after having a sense of dependence on Macao’s tangible resources, they have developed an intangible emotional identification. Consequently, they develop affective attitudes toward the place of study, including satisfaction with their life in Macao. Compared with tourists’ short-term travel satisfaction, the development of mainland Chinese students’ life satisfaction is longer. However, student life satisfaction is retained for longer, too. In addition, the formation of Mainland Chinese students’ life satisfaction is more complicated than the formation of tourists’ travel satisfaction. When they are living in Macau, they exhibit a long-lasting behavior change. Since lifestyle change can promote people’s wellbeing (Ramkissoon, 2020a,^2021^), so mainland Chinese students were satisfied with their quality of life. This study stimulates researchers’ interest in studying student life satisfaction and its consequences, such as students’ support for tourism development.

Second, this research links the theory of life satisfaction with the theory of place attachment to enrich the research of the factors influencing the potential for destination brand-building. Destination brand-building is behavioral tendencies related to place attachment. Individuals who are attached to a place can perceive that the place provides them with conditions to meet their action goals more than other places, so they are more likely to show a tendency to be close to the place. The results of this study reveal that place attachment, as a preliminary cognitive–emotional connection between sojourners and Macao, does not only turn to affective attitudes toward the place but also influences sojourners’ destination brand-building behaviors. The indirect effects of place dependence on destination brand-building behaviors (indirect effect PD → WOM = 0.449, indirect effect PD → AB = 0.418) are larger than its direct effect (direct effect PD → WOM = 0.201, direct effect PD → AB = 0.199), which implies that place identity and student life satisfaction acted as mediators complementing the effect of place dependence on destination brand-building. Using Mainland Chinese students as a case study, this research indicates the destination brand-building path for sojourners: place dependence → place identity → student life satisfaction → WOM and ambassador behavior. This study provides researchers with a research direction to conduct further studies in investigating destination brand-building behaviors for tourists, residents, and sojourners.

Given the uniqueness of sojourners travel in the host area and the increase in the number of sojourner groups (Liu and Ryan, 2011), this study also contributes to destination marketing research in understanding Mainland Chinese students’ (as sojourners’) perception of, and attitude to a destination. This study explores the destination brand-building path. Since as a group, sojourners also include working-holiday youngsters and deep-travel tourists, understanding their behavior is expected to provide meaningful implications for tourism planning. As some tourists tend to stay longer in a destination as they have more deeply understood a tourist destination’s culture (Brown, 2009), they become sojourners. This study explores a research target, for which the special market segment becomes more important in the further tourism market. Therefore, researchers should put more focus on sojourners.

In studying the behavior of ambassadors, researchers mainly focussed on local residents as the research objects because residents are internal stakeholders of the country; they are the majority of the people who live in the destination and the largest group that constitutes the destination brand. The active participation of residents in the formation and implementation of the brand is invaluable. In this process, they act as the city’s brand ambassadors (Konecnik Ruzzier and Petek, 2012; Wassler and Hung, 2017). Their brand citizenship behaviors can enhance the equity of a destination brand. However, for some tourist destinations, not only the residents of destinations are a legitimate and salient stakeholder group (Fyall and Garrod, 2019), sojourners are also important internal stakeholders. Compared with residents, the relationship between sojourners and potential tourists is closer. Since sojourners have lived in the destination for a long time, their promotion and guidance are considered more credible by potential tourists. Therefore, sojourners not only show internal brand behaviors but also exhibit external brand behaviors that affect the destination brand development. This study highlights the role of sojourners in enhancing the equity of a destination brand and fills the aforementioned theoretical gap so that people can understand more about this most complex group who are brand stakeholders of tourist destinations.

### Recommendations

From a practical point of view, this study provides more effective marketing strategies and programs for the governments, tourism agencies, and other stakeholders in destination countries. Firstly, the governmental tourism offices could utilize sojourners, especially Mainland Chinese students, to construct the destination brand. For example, tourism offices could launch the “Mainland college student tourism ambassador” scheme to recruit volunteers to participate in large-scale tourism promotion fairs in the cities of Mainland China. In this way, the volunteers could understand and become closer to the city where they study more and also have more responsibility for it.

Second, according to the internal brand and marketing approach, it is suggested that the higher education bureau could cooperate with universities and colleges to promote knowledge contests of the destination. For example, the universities in Macao could arrange a “Macao historical knowledge competition” or a “Best Macao Photography Contest” for Mainland Chinese students to create a uniquely positive experience of Macao. These activities help to establish the Mainland Chinese students’ emotional connection with Macao, so as to motivate them to become actively involved in the construction of the Macao destination brand.

Third, dependence on local facilities and resources is the basis of the formation of personal life satisfaction, so governments and other stakeholders should take a leading role in improving sojourners’ life satisfaction. For example, universities and colleges in Macao could establish a “Senior Supervisor” plan to help first-year Mainland Chinese students to familiarize themselves with campus life and the living environment in Macao.

From the perspective of the tourism market, Mainland Chinese students have a direct and indirect role in destination marketing. They act as an attraction factor to motivate their friends and family to visit. Therefore, the Macao government tourism office could cooperate with Macao airlines, hotels, and other related industry organizations to provide promotional packages for Mainland Chinese students and their families. For example, these could be “back to school after vacation preferential ticket counterparts” and “preferential hotel discount coupons,” which would help to develop Mainland Chinese students’ place attachment and increase their life satisfaction. It would also help to increase tourism revenues.

### Limitation

The limitations of this study should be acknowledged, and this opens opportunities for future research. First, there are regional limitations in the collection of sample data. The severe impact of the COVID-19 epidemic has caused a restriction on Mainland Chinese students returning to study in Western countries (United States and United Kingdom). The collection of samples in other countries is recommended in order to obtain results that can be generalized. Second, this study only provides a basic model for studying sojourners’ behaviors in the light of place attachment theory and life satisfaction theory, and future studies could be extended on the basis of this research model. Third, from the perspective of research objects, the sojourners involved in this study are Mainland Chinese students, while for some countries, such as Singapore, the labor group accounts for a large part of sojourners. Therefore, attention should be paid to including the labor group in the follow-up research.

## Data Availability Statement

The original contributions presented in the study are included in the article/supplementary material, further inquiries can be directed to the corresponding author/s.

## Ethics Statement

Ethical review and approval was not required for the study on human participants in accordance with the local legislation and institutional requirements. Written informed consent from the participants to participate in this study was not required in accordance with the national legislation and the institutional requirements.

## Author Contributions

XW contributed to the development of the research framework, the supervision of research, and the revision of the manuscript. IKWL directed the writing of the manuscript, performed data analysis, and revised the manuscript. XYL was responsible for collecting data, drafting the manuscript, revising the manuscript, and managing manuscript submission. All authors contributed to the article and approved the submitted version.

## Conflict of Interest

The authors declare that the research was conducted in the absence of any commercial or financial relationships that could be construed as a potential conflict of interest.

## Publisher’s Note

All claims expressed in this article are solely those of the authors and do not necessarily represent those of their affiliated organizations, or those of the publisher, the editors and the reviewers. Any product that may be evaluated in this article, or claim that may be made by its manufacturer, is not guaranteed or endorsed by the publisher.
